# Necroptosis: A new target for prevention of osteoporosis

**DOI:** 10.3389/fendo.2022.1032614

**Published:** 2022-10-19

**Authors:** Xinli Hu, Zheng Wang, Chao Kong, Yu Wang, Weiguo Zhu, Wei Wang, Yongjin Li, Wei Wang, Shibao Lu

**Affiliations:** ^1^ Department of Orthopedics, Xuanwu Hospital, Capital Medical University, Beijing, China; ^2^ National Clinical Research Center for Geriatric Diseases, Xuanwu Hospital, Capital Medical University, Beijing, China

**Keywords:** osteoporosis, necroptosis, cell death, osteoclasts, osteoblasts

## Abstract

Multiple causes may contribute to osteoporosis, characterized by a loss in bone mass and density as a consequence of the degradation of bone microstructure and a resultant rise in bone fragility. Recently, increasing attention has been paid to the role of necroptosis in the development of osteoporosis. Necroptosis is orchestrated by a set of proteins known as receptor-interacting protein kinase (RIPK)1, RIPK3, and mixed lineage kinase domain-like protein (MLKL). A necrosome is formed by MLKL, RIPK1, RIPK3, and RIPK3-RIPK3. A dissociated MLKL forms pores in the plasma membrane and eventually leads to necroptosis after translocating from the necrosome. In this review, we discuss a detailed understanding of necroptosis and its associated processes, a better understanding of its interactions with osteoclasts, osteoblasts, and osteocytes, and the associations between necroptosis and diabetic osteoporosis, steroid-induced osteoporosis, and postmenopausal osteoporosis. In addition, a variety of experimental medicines capable of modulating crucial necroptosis processes are highlighted. It’s important to note that this is the first review paper to consolidate current data on the role of necroptosis in osteoporosis, and it offers fresh hope for the future treatment of this disease.

## Introduction

Micro-architecture of bone tissue degeneration and reduced bone mass define osteoporosis (OP), a systemic disease (1). Osteoporosis affects over 200 million individuals worldwide, and the number continues to rise yearly. In particular, hip fractures, vertebral fractures, and distal forearm fractures are at an increased risk due to increased bone fragility (2). The most common cause of fracture in elderly individuals is osteoporosis, which accounts for around 9.0 million fractures annually (3). A considerable amount of pain and suffering is involved in fragility fractures, as well as disability, death, and societal costs associated with these injuries. The bone is constantly resorbed and reformed according to a process called remodeling, controlled by two major cell types: osteoclasts and osteoblasts (4). Osteoporosis (OP) is caused by an imbalance in bone metabolism. Osteoporosis occurs when bone resorption outpaces bone creation, which may result from normal aging or other pathological diseases (5). Currently, the treatment strategies for OP mainly use drug therapy, including calcitonin, bisphosphonates and fluorides (6, 7). These drugs may relief some of the bone loss and clinical symptoms. However, their long-term clinical efficacy is limited. Because they have poor tolerance, significant side effects and high cost (8, 9). All these reasons making the development of new, safe and effective OP treatment is imperative.

Programmed cell death with a necrotic appearance is called necroptosis and occurs in several biological processes, including inflammation, the immunological response and metabolic disorders (10).,RIPK1 (receptor-interacting protein kinase 1), RIPK3 (receptor-interacting protein kinase 3), and MLKL (mixed-lineage kinase domain-like pseudokinase) play an important role in this process (11). Studies have shown that death receptors such as tumor necrosis factor receptor 1 (TNFR1), Fas ligand receptor (FasR), TNF-related apoptosis-inducing ligand receptor (TRAIL-R), interferons (IFNs), intracellular RNA and DNA sensors, and other mediators are effective in inducing necroptosis (12, 13). Among these signaling, TNFR1 signaling is best characterized and believed to be extensively involved in necroptosis (14).

**Figure 1:**
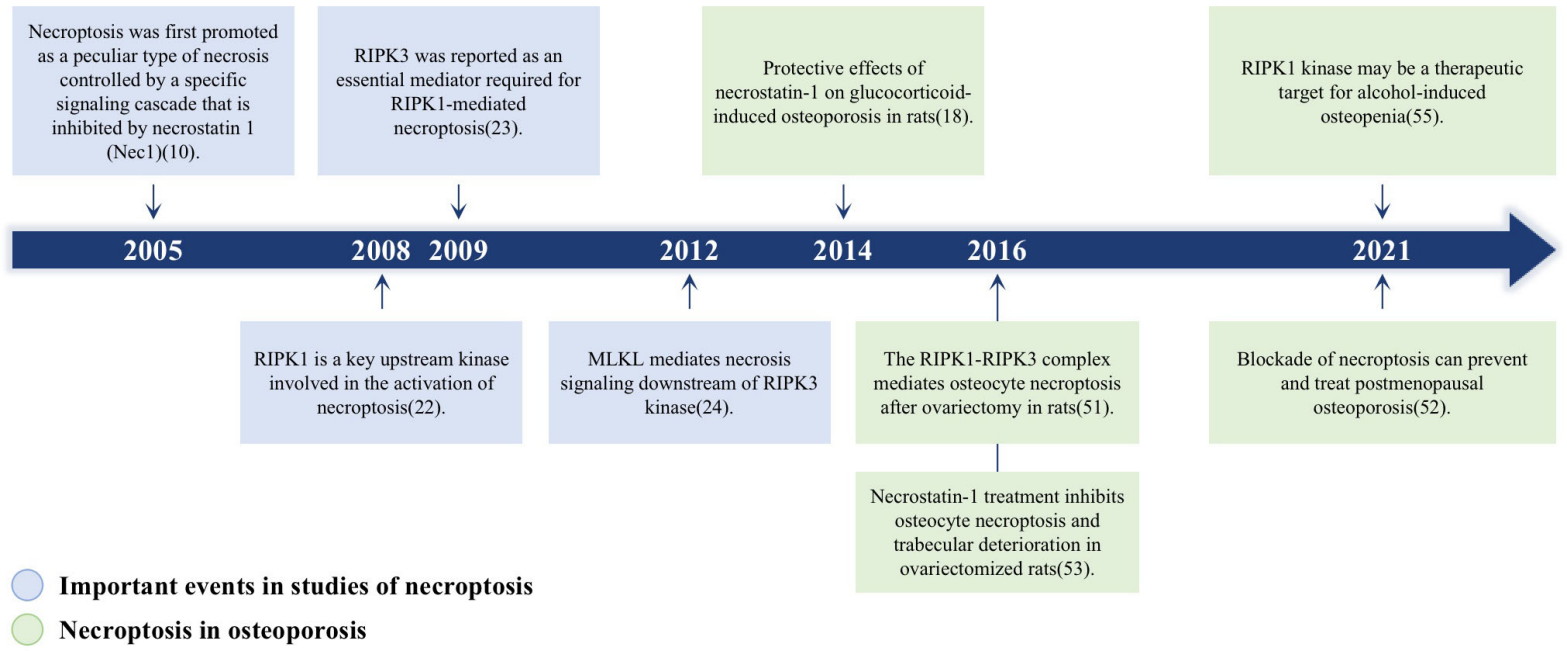
A timeline of the momentous events for necroptosis and its role in osteoporosis. Blue boxes highlight the major findings in the discovery of necroptosis signaling pathway; Green boxes represent the researches about the role of necroptosis in osteoporosis.

The association between necroptosis and osteoporosis has increased in recent studies. We draw a **Figure 1** to illustrate the momentous events for necroptosis and its research in osteoporosis. One of the most common types of osteoporosis in women is postmenopausal osteoporosis (PMOP), which arises after the cessation of estrogen (E2) in women. Ovarian ovariectomized rats in several studies had higher levels of tumor necrosis factor-alpha (TNF-α) when estrogen was deficient (15, 16). Moreover, TNF-α is an essential cytokine that activates TNFR1 and induces necroptosis (17). Necroptosis hence may have a significant role in PMOP. In addition, rats with glucocorticoid-induced osteoporosis who were administered necroptosis inhibitor (Nec-1) had improved bone production (18). These findings may indicate that necroptosis is intimately associated with osteoporosis. Here, we summarize the basic pathological features of necroptosis, the relationship between necroptosis and osteoclasts and osteoblasts as well as the relationship between necroptosis and osteoporosis. We also illustrate how necroptosis influences diabetic osteoporosis (DOP), glucocorticoid-induced osteoporosis (GIOP), and postmenopausal osteoporosis (PMOP).

## Overview of necroptosis

According to the morphological and biochemical changes of cell death. The Nomenclature Committee on Cell Death (NCCD) roughly classifies cell death into three categories: apoptosis, autophagy and necrosis (19, 20). Apoptosis is the first type of cell death, characterized by small cells, cell membrane blebbing, nuclear fragmentation and chromatin concentration (21). Two apoptosis pathways have been found in organisms, including the intrinsic and the extrinsic apoptotic pathway. Its biochemical characteristics and mechanism are defined as cascading by the activation of caspases-8 or 9 and subsequent activation of caspases-3/6/7 (22). The second type of cell death is autophagy, which is characterized by the accumulation of double membrane covered vacuoles covering cytoplasm or cytoplasmic organelles, and the redistribution of light chain 3 (LC3) to autophagy membranes (19). Autophagy-related (ATG) families, mammalian target of rapamycin (mTOR), LC3-I, LC3-II and p62 are involved in autophagy (23). Necrosis, as the third type of cell death, is characterized by the rupture of the plasma membrane and release of cell contents (24). The release of damage associated molecular model (DAMP) and cell contents provides feedforward signals to enhance programmed necrosis in other cells (25). The distinctive features among these different death modes are listed in **Table 1**. Most studies of osteoporosis focus on apoptosis and autophagy, because necrosis has long been considered irreversible (26, 27). However, recently, more and more evidence has revealed a special form of necrosis, called necrotic apoptosis, involving osteoporosis (28, 29). First, A distinct signaling cascade blocked by necrostatin-1(Nec1) governs necroptosis, described in 2005 by Degterev and colleagues (10). The term necroptosis was first used in 2018, in reference to a type of programmed necrosis not triggered by cysteine-aspartic proteases (caspases) and governed mainly by two receptor interacting protein kinases: RIPK1 and RIPK3 (19). Necroptosis is a newly found mechanism of intentional cell death that has a distinct morphology from apoptosis. It is characterized by plasma membrane rupture, cell content leaking, and organelle enlargement (30, 31). Necroptosis is not reliant on caspase, but is regulated by various cellular signal transduction proteins, such as RIPK1, RIPK3, and MLKL (32–34). In research studies, several cell mediators have been demonstrated to trigger the necroptosis pathway, such as members of the tumor necrosis factor (TNF) family, Fas ligands, interferon, and RNA or DNA sensors (35–37). Death receptor ligands, particularly TNF and FAS, are the most well-studied inducers of necroptosis (19). Once TNF interacts with TNFR1, TNFR1 recruits TRADD, TRAF2/5, cIAP1/2, LUBAC, and RIPK1 to form the so-called complex I, which activates NF-kB and its transcription of pro-survival and pro-inflammatory genes (37). Deubiquitination of RIPK1 by cylindromatosis (CYLD) and ubiquitin- editing enzyme A20(A20) can result in RIPK1 dissociating from complex I; then, the remaining complex recruits TRADD, FADD and caspase-8 and forms complex IIa (composed of TRADD, Fas-associated death domain (FADD), RIPK1 and caspase-8), which activates apoptosis (38, 39). At the same time, deubiquitination also result in complex I transformed into complex IIb to induce necroptosis, which consists of RIPK1, RIPK3, Fas-associated death domain protein (FADD), and caspase-8 (38, 40). It means that the deubiquitination of complex I transforms into complex II (IIa and IIb). Two distinct types of complex II can be distinguished based on their composition and the activity of their proteins.

**Table 1:** The distinctive morphological and main biochemical features of apoptosis, autophagy, and necrosis.

Type of cell death	Morphological features			Main biochemical features
	Plasma membrane	Nucleus	Cytoplasm	
Apoptosis	Membrane blebbing	Nuclear fragmentation	Shrinkage	Activation of caspases-3/6/7/8/9
Autophagy	Membrane rupture	Minor changes	Vacuolation	ATG family of gene encoded proteins, LC3 conversion and cleavage of p62.
Necrosis	Membrane rupture	Dilatation of nuclear membrane	Translucence	Release of DAMPs and cell contents

**Figure 2:**
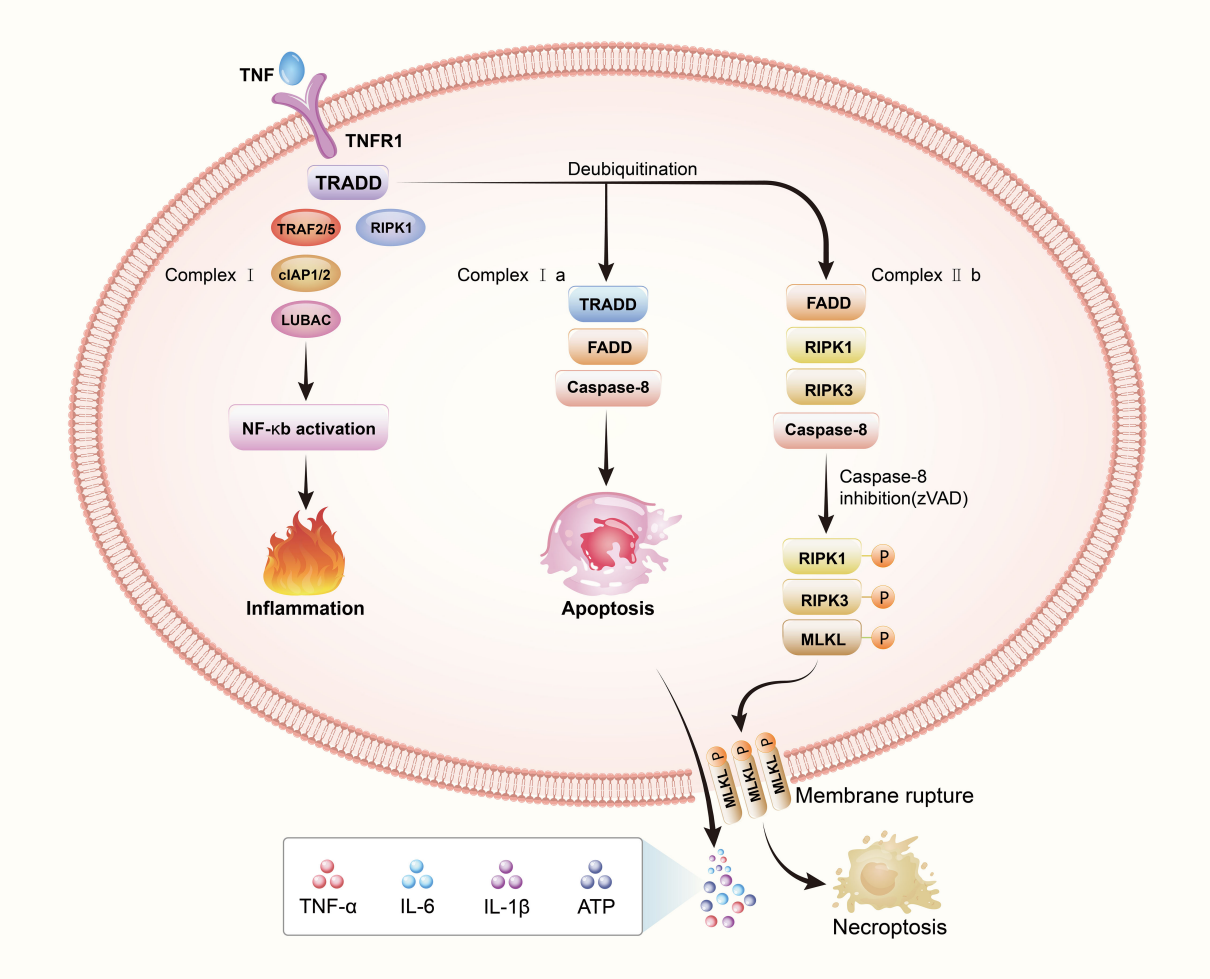
Necroptosis: a regulatory cell death. The death receptor signaling pathway is responsible for necroptosis This image depicts the interaction between tumor necrosis factor (TNF) and TNF receptor 1(TNFR1). When the binding of tumor necrosis factor-α(TNFα) to TNF receptor 1 (TNFR1), TNFR1 ligation triggers the assembly of complex I composed of TRADD, RIPK1, TRAF2/5, cIAP1/2 and LUBAC. Cell survival, apoptosis, and necroptosis are all possible consequences of complex I formation, which are regulated by different downstream cytosolic signaling complexes. Cell survival is boosted by ubiquitination of RIPK1, which activates the NF-κB pathway and promotes cell proliferation. Under a certain circumstance, deubiquitination of RIPK1 can result in the complex recruiting TRADD, FADD and caspase-8 forming complex IIa which activates apoptosis, or recruiting FADD, RIPK3, and caspase-8 forming complex IIb, which activates necroptosis. By blocking caspase-8, the binding of activated RIPK1 and RIPK3 leads to RIPK3 activation, which then phosphorylates and activates a pseudokinase called MLKL. MLKL undergoes oligomerization and translocates to and permeabilizes the plasma membrane to induce necroptosis.

To summize, complex I is pro-survival, complex IIa is pro-apoptotic, and complex IIb is pro-necroptotic (19). The type of complex II (a or b) and the state of caspase 8 (activated or inactivated) determine the transition to apoptosis or necroptosis (41). RIPK3, considered one of the key regulators of necroptosis, is required for necroptosis (42). A necrosome is formed when complex IIb recruits substantial quantities of RIPK3 in the presence of high levels of this protein (43). RIPK1 and RIPK3 are degraded by caspase-8 in the necrosome, preventing necroptosis (37). When zVAD inhibits caspase-8, RIPK1 recruits RIPK3 and phosphorylates RIPK3 in response (33). A phosphorylated RIPK3 recruits MLKL and phosphorylates MLKL, and then MLKL occurs oligomerization and translocation into the plasma membrane. Exactly how MLKL causes necrosis is still a mystery, although there are two competing hypotheses at the moment: It’s possible that MLKL serves as a plasma membrane platform for the recruitment of Ca^2+^ or Na^+^ ion channels (44, 45), or that MLKL forms part of a complex that creates a pore in the membrane (46). In order to better understand the process of necroptosis, we draw a **Figure 2** to show the process.

## Necroptosis with bone cells

A bone cell consists of osteocytes, osteoclasts, and osteoblasts. Adult skeletons are composed primarily of osteoclasts. A large percentage of bone cells are comprised of osteocytes (90-95%), while a smaller percentage is comprised of osteoclasts and osteoblasts (5-10%) (47). A bone mesenchymal stem cell (BMSC) differentiates into osteoblasts and osteocytes (48). Osteocytes become encased in their surrounding recently-mineralized bone matrix and then the new bone is formed (49, 50). The bone remodeling process and the incidence of osteoporosis are influenced by osteoocytes, osteoclasts, and osteoblasts (51). Maintaining a healthy balance between bone formation and destruction, as well as a continuous cycle of bone remodeling, are essential for maintaining the integrity of bone tissue. Osteoclasts mainly play the role of bone resorption, whereas osteoblasts mainly play the role of bone reconstruction, including the formation, mineralization, and secretion of osteocytes. They mutually restrict and balance the metabolism of bone tissues (52, 53). When an imbalance appears between the bone resorptive and bone-forming, this imbalance leads to loss of bone mass and strength, resulting in osteoporosis (54, 55). Obsession with the link between necroptosis and osteoporosis has grown during the past two decades. Necroptosis, which occurs in bone cells, such as osteocytes, osteoclasts and osteoblasts, has been linked to bone loss.

## Necroptosis occurs in osteocytes

Approximately 90% to 95% of the bone’s mature cells are osteocytes (56, 57). Osteocytes play a vital function in transmitting diverse signals to the interfaces between osteoclasts and osteoblasts (58), demonstrating that osteocytes may engage in the regulation of bone resorption and creation (58). In addition, mounting evidence is that the continuing loss of osteocytes due to cell death significantly contributes to osteoporosis (59, 60). It was discovered in 2016 by Cui et al. that ovariectomy (OVX) rats suffering from estrogen deficiency had higher rates of osteocyte apoptosis and necroptosis (29). Nec-1 (a particular inhibitor of receptor-interacting protein 1) was shown to prevent significantly necroptotic death of osteocytes, thereby slowing down the loss of bone mass in the animals. However, it has not been shown to a considerable degree that necroptosis and apoptosis are responsible for the loss of osteocytes, however. That’s why the study of necroptosis and apoptotic cell death in rats with osteoporosis deficiency has been extended by He et al. (60). Apoptosis and necroptosis were found to contribute to osteocyte loss in OVX-induced osteoporosis, with necroptosis having a more significant influence than apoptosis on osteocyte loss. There is a strong correlation between necroptosis and osteoporosis development based on these findings.

## Necroptosis occurs in osteoblasts

One of the most significant aspects of bone regeneration is osteoblasts, which play a crucial role in a bone matrix formation, secretion, and mineralization (61). Increased necroptosis of osteoblasts and reduced bone formation and osteogenesis were seen in a model of excessive ethyl alcohol consumption in mice, as shown by Guo et al. in their study. A key inhibitor of RIPK1 kinase in the necroptosis pathway, necrostatin-1 (Nec-1), inhibits RIPK1/RIPK3/MLKL signaling, and thus might inhibit alcohol-induced osteopenia through decreased activation of osteoblast necroptosis (28). Furthermore, Shi et al. discovered concurrently identical events implicating necroptosis in the cell death of mouse osteoblasts (62).

## Necroptosis occurs in osteoclasts

In the bone-resorbing pit between the osteoclasts and the bone, The Osteoclasts secrete lysosomal enzymes to degrade the mineralized bone matrix and perform the function of bone resorption (63). The osteoclastogenesis process depends on transforming growth factor (TGF-)-activated kinase 1 (TAK1). Loss of TAK1 potentiates spontaneous necroptosis in osteoclasts. It suggests necroptosis occur in osteoclasts (64). Inhibiting RIPK1 ubiquitination occurs using Smac-mimetics (SM), which activates RIPK1 and causes necroptosis. After application of the SM, human osteoclasts showed less stability and viability, and induced TNF-dependent cell death in osteoclasts precursors (pre-osteoclasts) (65). It shows that necroptosis inhibits human osteoclastogenesis, and that the regulatory mechanism may provide interesting avenues for lowering bone deterioration. Mullinet et al. proved, from the standpoint of genetic gene variations, that RIPK3 is one of the genes identified in the research as associated with osteoporosis (66). In RIPK3-deficient mice, the degree of diaphyseal trabecularisation was observed to be drastically reduced. Analysis of bone sections stained with TRAP indicated a substantial increase in the overall number of osteoclasts in the bones of RIPK3-deficient animals. An excess of osteoclasts might result from the absence of RIPK3, which is involved in osteoclast necroptosis. Loss of necroptosis may lead to an excess of osteoclasts. Ultimately contribute to significant bone loss.Following the aforementioned research, we assumed that necroptosis of osteoclasts would reduce the occurrence of bone resorption and increase bone formation. In contrast, necroptosis of osteoblasts and osteocytes would lead to reduced bone and bone formation.

## Potential relationship between necroptosis and osteoporosis

**Figure 3:**
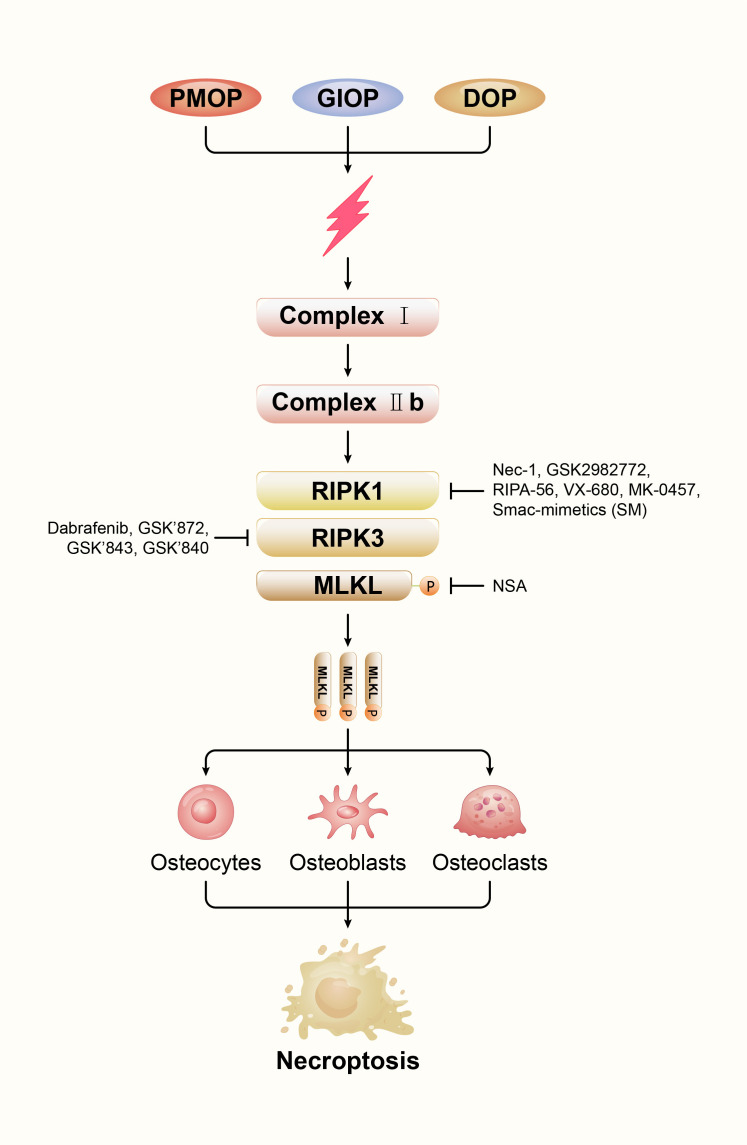
Effects of various therapeutic inhibitors of necroptosis on components of necrosomes.

As a result of osteoporosis, bone mass declines, along with its microarchitecture, making the bones more fragile and more likely to break (67). OP is classified as primary, secondary, and idiopathic. Senile osteoporosis (SOP) and postmenopausal osteoporosis (PMOP) are the most common forms of OP. Age-related bone loss is the hallmark of SOP. In general, it refers to osteoporosis after 70 years of age and is caused by specific biological aging of the skeletal system (68). Another primary osteoporosis is PMOP, mainly due to estrogen deficiency. The most common secondary osteoporosis is diabetic osteoporosis (DOP) and glucocorticoid-induced osteoporosis (GIOP) (69). The necroptosis in osteocytes, osteoblasts and osteoclasts and the imbalance of bone metabolism are involved in the pathophysiological process of primary and secondary osteoporosis. In order to better understand the relationship between necroptosis and osteoporosis, we draw a **Figure 3** to show their relationship.

## Necroptosis and PMOP

One of the most common types of osteoporosis in women is PMOP, which arises after the cessation of estrogen (E2) in women. Customarily, the twin processes of bone resorption and creation are closely connected to preserving bone homeostasis (70). With PMOP, postmenopausal women have a certain degree of bone loss, resulting in a shift toward higher bone resorption and reduced bone growth (71). Bone cell apoptosis and necroptosis have been substantially linked to PMOP (51, 66). In addition to apoptosis, OVX-induced estrogen deficiency in the rat femur enhanced necroptosis in osteocytes, according to Cui et al. Osteocyte necroptosis is shown to be a substantial contributor to estrogen deficiency-induced bone loss in the OVX rat model (59). As far, as the roles of necroptosis and apoptosis in osteocyte loss remain largely a scientific mystery. When estrogen shortage is present, necroptosis and apoptosis play a significant role in the gradual loss of osteocytes (60). Their findings suggest that OVX-induced osteoporosis is caused by necroptosis, which may have a greater impact on osteoclast loss than apoptosis, and apoptosis, which is thought to have a less impact on osteoclast loss. Nec-1 therapy also significantly reduces necroptosis in OVX mice, therefore preventing osteoporosis development, according to Cui et al. Because of this, scientists believe that the death process known as necroptosis might be a unique method of treating osteoporosis (29).

## Necroptosis and GIOP

GIOP has become the most common form of secondary OP in recent years, and it is commonplace in adolescents and young adults (72). The suppression of bone growth by GCs has been regarded as a crucial factor in this occurrence, the fact that the specific processes behind GIOP are still unclear. A previous study reported that apoptosis is an essential mechanism in the GIOP process (73). However, the modest amount of apoptotic osteoblasts cannot explain the elevated bone fragility observed in GC-treated patients (74). It might suggest that GIOP involves other cell death pathways in osteocytes and osteoblasts. For the first time, Feng et al. are examining the expression of RIPK1 and RIPK3 in GIOP rats after glucocorticoid (dexamethasone) injection to induce osteoporosis pharmacologically (18). Nec-1 protect GIOP rats is then tested utilizing biochemical markers and histomorphometric indices. When given necroptosis inhibitor Nec-1, rats suffering from glucocorticoids produces more bones because of the necroptosis inhibitor Nec-1, the researchers found. This evidence has indicated that necroptosis play an important role in the progression of GIOP. Thus, more studies are required to better understand the processes of necroptosis in GIOP.

## Necroptosis and DOP

By 2045, 578 million and 700 million persons are expected to live with diabetes, respectively. Diabetes is expected to affect one in every eleven adults in 2019, or 463 million (75). The condition of DOP occurs when there is a decrease in bone mineral density (BMD) and a change in bone microstructure, as well as an increase in bone fragility due to diabetes. A significant financial burden is imposed on patients suffering from DOP, which significantly diminishes their quality of life (76). On a yearly basis, DOP rates rise as diabetes prevalence increases around the world. There is evidence from clinical studies that between 50 and 65% of people with diabetes have lower bone mineral density and are at a higher risk of fractures, while roughly 35% have osteoporosis (77). The exact processes of DOP are still unclear. Loss of osteocyte viability has been proposed as a major pathogenic component in DOP (78). While it is yet unknown how osteocytes occurs cell death, Some studies show that apoptosis, autophagy, and ferroptosis are significant mechanisms of osteocyte death in DOP (78–80). However, whether necroptosis is involved in DOP has not been experimentally confirmed. Among other complications of diabetes, such as diabetic nephropathy, diabetic retinopathy, and diabetic cardiomyopathy, many studies have reported that necroptosis has an important role (81–83). Therefore, we speculate that necroptosis also has a role in DOP. This will be an interesting direction for future research.

## Potential therapeutic strategies for necroptosis

A specific group of protein molecules mediates necroptosis. Therefore, inhibitors have been developed by researchers to target these key proteins. We described the effects of various inhibitors on necroptosis, with the aim of identifying new and therapeutic agents for necroptosis (**Figure 3**). These findings of necroptosis inhibitors will provide potential treatment strategies for osteoporosis.

### RIPK1 inhibitors

Degterev et al. in 2005 demonstrated a particularly potent small-molecule necrosis inhibitor called Nec-1 (10). The mechanism of action of Nec-1 is its binding to the hydrophobic pocket of the carboxyl and amino lobes of the RIPK1 kinase domain (32, 84). Cui et al. investigated that Nec-1 can prevent necroptotic osteocytes, mediated by RIPK1, and inhibit osteoporosis progression in OVX rats (29). In addition to Nec-1, GSK2982772, compound 56 (RIPA-56) and VX-680 and MK-0457 were confirmed as emerging inhibitors of RIPK1 (85–87). These drugs have been used in other diseases and have potential for the treatment of pathology caused or aggravated by necroptotic cell death. However, its application in osteoporosis still needs to be further explored. Collectively, RIPK1 kinase may serve as a new target for the development of therapeutic drugs for osteoporosis.

### RIPK3 inhibitors

RIPK3 is a critical regulator of necroptosis. The B-Raf inhibitor dabrafenib was demonstrated to be a potent inhibitor of RIPK3 and is used in the treatment of melanoma (88). The mechanism of dabrafenib is to disrupt the interaction between RIPK3 and MLKL (89). In mouse models of ischemic brain injury, Dabrafenib may attenuate TNF-α-induced necroptotic pathway (90). However, whether dabrafenib can inhibit necroptosis and delay the progression of osteoporosis remains to be determined. In addition, GSK’872, GSK’843 and GSK’840 were identified as a class of specific RIPK3 inhibitors of necroptosis by Mocarski and colleagues (91). The application of these drugs to osteoporosis remains to be explored.

### MLKL inhibitors

The phosphorylation of MLKL has a significant role in regulating necrosis. However, few effective inhibitors have yet been identified. Hildebrand et al. reported a small molecule (unnamed) that binds the MLKL pseudokinase domain and retards membrane translocation to inhibit necroptotic signalling (92). In addition, Necrosulfonamide (NSA) inhibits MLKL-Mediated necrosis by blocking its N-Terminal CC domain function of MLKL (34). Considering that MLKL is an essential protein for the execution of necroptosis, it may be a potential ideal target for inhibiting necroptosis.

## The future of necroptosis discussion and perspectives

Deficiencies in bone density and microstructure are hallmarks of osteoporosis, a systemic metabolic bone disease (93). Osteoporosis and its debilitating effects continue to pose difficulties for clinicians. Therefore, understanding the molecular mechanisms of necroptosis and osteoporosis can provide substantial insights into the field of bone metabolism.

Necroptosis and osteoporosis are discussed in this article, specifically PMOP, GIOP, and DOP. Although tremendous progress has been made in the research of necroptosis, several challenges need to be resolved. Necroptosis research is still in its infancy, and its precise mechanism and associated signalling pathways are yet unknown. To create successful treatments, it is necessary to investigate these newly identified processes, their interconnected signalling networks, and molecular targets. An effective method of treating osteoporosis is to use Nec-1, an inhibitor of necroptosis that has been demonstrated to be a specific inhibitor of necroptosis (18, 29). Studies on the pharmacologic inhibition of necroptosis in osteoporosis is limited. Therefore, it’s necessary to found other potential drugs to depress necroptosis in osteoporosis. In addition, the role of necroptosis has been confirmed in castrated mice and glucocorticoid mice. However, in diabetic mice, it has not yet been confirmed, so it is necessary to confirm the relationship between osteoporosis and necroptosis in diabetic mice.

Currently, there is a lot of interest in finding out more about the processes that underlie necroptosis and osteoporosis thanks to these promising new paths in study. We have a few ideas for further research: (1).Bone reconstruction is orchestrated by osteoblast, osteocyte and osteoclast mutually (94). The interaction and crosstalk among osteoclasts, osteoblasts, and osteocytes should be considered in the treatment of osteoporosis, especially when osteoclasts, osteoblasts, and osteocytes undergo necroptosis. (2) One research has systematically confirmed that the necroptosis pathway is involved in the iron overload-induced death of osteoblastic cells for the first time. ROS as its key molecule (95). This suggests a link between necroptosis and ferroptosis. Moreover, cell death includes autophagy, pyroptosis, ferroptosis, necroptosis, and lysosomal cell death. So are these types of cell deaths having a connection with osteoporosis? Are there interactions and crosstalk between these cell deaths in osteoporosis? Thus, further researches are needed to be comprehensively investigated the correlation among autophagy, necroptosis, ferroptosis, pyroptosis and lysosomal cell death in osteoporosis. (3).it is essential to elucidate which type of programmed cell death plays the dominant role in osteoporosis

## Conclusion

Necroptosis is a regulated programmed cell death involved in developing and progressing many diseases. Osteoporosis is attracting more and more attention because of its high morbidity. An overview of necroptosis and its relevance to osteoporosis is provided in this review. A better understanding of necroptosis may generate new ideas for bone metabolism research. Osteoporosis medications now on the market target osteoclasts, which are responsible for bone resorption. These medications can slow the growth of osteoporosis, but they also have some unwanted side effects. Necroptosis seems to have a therapeutic impact on osteoporosis by influencing osteoclasts, osteoblasts, and osteocytes, according to this promising new study. It is predicted to be a novel treatment for osteoporosis prevention and treatment with fewer adverse effects as a result of necroptosis management. Despite necroptosis being a unique kind of cell death, additional research is needed to uncover new targets for controlling necroptosis in the therapy of osteoporotic bone loss, which might lead to more effective and better therapeutic alternatives.

## Author contributions

XH and ZW searched and reviewed literature, drafted manuscript and revision; CK and YW discussed and revised the manuscript; WZ and WW (8th author) and YL provided critical comments; WW (8th author) and SL designed and formulated the review theme, revised and finalized the manuscript.

## Funding

This study was supported by the National Key Research and Development Program of China (No.2020YFC2004900), National Natural Science Foundation of China (H0608), Natural Science Foundation of China (No.81672201, 81871794) and Beijing Hospitals Authority’Ascent Plan (No.DFL20190802).

## Conflict of interest

The authors declare that the research was conducted in the absence of any commercial or financial relationships that could be construed as a potential conflict of interest.

## Publisher’s note

All claims expressed in this article are solely those of the authors and do not necessarily represent those of their affiliated organizations, or those of the publisher, the editors and the reviewers. Any product that may be evaluated in this article, or claim that may be made by its manufacturer, is not guaranteed or endorsed by the publisher.
